# Radiological data of brachial plexus avulsion injury associated spinal cord herniation (BPAI-SCH) and comparison to anterior thoracic spinal cord herniation (ATSCH)

**DOI:** 10.1016/j.dib.2020.105333

**Published:** 2020-02-27

**Authors:** Andrew S. Jack, Jens R. Chapman, Praveen V. Mummaneni, Carter S. Gerard, Line Jacques

**Affiliations:** a Department of Neurological Surgery, University of California San Francisco (UCSF), 400 Parnassus Ave., Eighth Floor, San Francisco, CA, 94122, USA; b Swedish Neuroscience Institute (SNI), Swedish Medical Center, 550 17th Ave #540, Seattle, WA, 98122, USA

**Keywords:** Spinal cord herniation, Nerve root avulsion, Brachial plexus injury, Complication, Pseudomeningocoele

## Abstract

Spinal cord herniation (SCH) is a rare cause of myelopathy. When reported, SCH has most commonly been described as occurring spontaneously in the thoracic spine, and being idiopathic in nature (anterior thoracic spinal cord herniation, ATSCH) [1–3]. Several theories have been proposed to explain its occurrence, including congenital, inflammatory, and traumatic etiologies alike [1–4]. Even more rarely, SCH has been described to occur in the cervical spine in association with brachial plexus avulsion injuries (BPAI-SCH). In our accompanying article, “Late Cervical Spinal Cord Herniation Resulting from Post-Traumatic Brachial Plexus Avulsion Injury,” two cases of BPAI-SCH are presented and discussed in the context of the reviewed literature [5]. Here, pertinent accompanying follow-up data was collected and is presented for the cases, including postoperative radiographic outcome imaging. Furthermore, a table is presented comparing and contrasting ATSCH to BPAI-SCH. Although the two phenomena have been previously grouped together, this table highlights ATSCH and BPAI-SCH as distinct entities; more specifically, BPAI-SCH is a separate, long-term complicating feature of BPAI. This supplementary data helps treating physicians by increasing awareness and knowledge of BPAI-SCH as a distinct entity from ATSCH and cause of delayed neurological deterioration.

## Highlights

• Myelopathy due to spinal cord herniation (SCH) is rare.
• Brachial plexus avulsion injury associated SCH (BPAI-SCH) is a unique type of SCH.
• Follow-up data for a case of BPAI-SCH treatment is presented.
• BPAI-SCH is compared and contrasted with more common SCH subtypes.
• BPAI-SCH recognition and treatment is essential for patient improvement.

**Table:** Specifications Table

Subject	-Medicine and Dentistry
Specific subject area	-Surgery
Type of data	-Table and Figure
How data were acquired	-Magnetic resonance imaging (MRI) scanner -Computed tomography (CT) scanner -Plain radiograph (XR) -Literature review
Data format	-Raw and analysed supplementary figure and table to accompany research article.
Parameters for data collection	-Patient demographic, clinical and radiographic information was collected from an electronic database. -Literature review: PubMed, Ovid-Medline, and Google Scholar databases
Description of data collection	Patient demographic, clinical, and radiographic information was collected from an electronic medical database during routine patient care for postoperative case follow-up. PubMed, Ovid-Medline, and Google Scholar databases were searched from inception to 2019 for clinical articles in English related to spinal cord herniation. Articles were reviewed for ATSCH and BPAI-SCH data pertaining to demographic, clinical, radiographic, and technical features for the two entities. Patient postoperative imaging as part of their routine care was used for figure construction and features outlined above from the literature review were used for supplementary table construction.
Data source location	Patient care and imaging were completed at the University of California San Francisco, San Francisco, California, USA and the Swedish Medical Center, Seattle, Washington, USA.
Data accessibility	-With the article
Related research article	Author's name: Andrew S Jack, MD, MSc, FRCSC, Jens R Chapman, MD, Praveen V Mummaneni, MD, Line Jacques, MD, MSc, FRCSC Carter S Gerard, MD, Title: Late Cervical Spinal Cord Herniation Resulting from Post-Traumatic Brachial Plexus Avulsion Injury Journal: World Neurosurgery DOI: In Press

**Table:** 

**Value of the Data** • The data highlights Brachial Plexus Avulsion Injury Associated Spinal Cord Herniation (BPAI-SCH) as a rare, reversible, cause of myelopathy. • The data emphasizes BPAI-SCH as being distinct from other variations of SCH. • The data benefits physicians treating these patients (ie: neurosurgeons, spine surgeons, peripheral nerve surgeons). • Further knowledge and awareness of BPAI-SCH may lead to more prompt diagnosis and treatment for these patients. • Increased BPAI-SCH recognition may increase its epidemiological and pathophysiological research. • Recognition/treatment of this delayed cause of neurological deterioration is crucial for patient improvement.

## 1 Data description

**Fig. 1:**
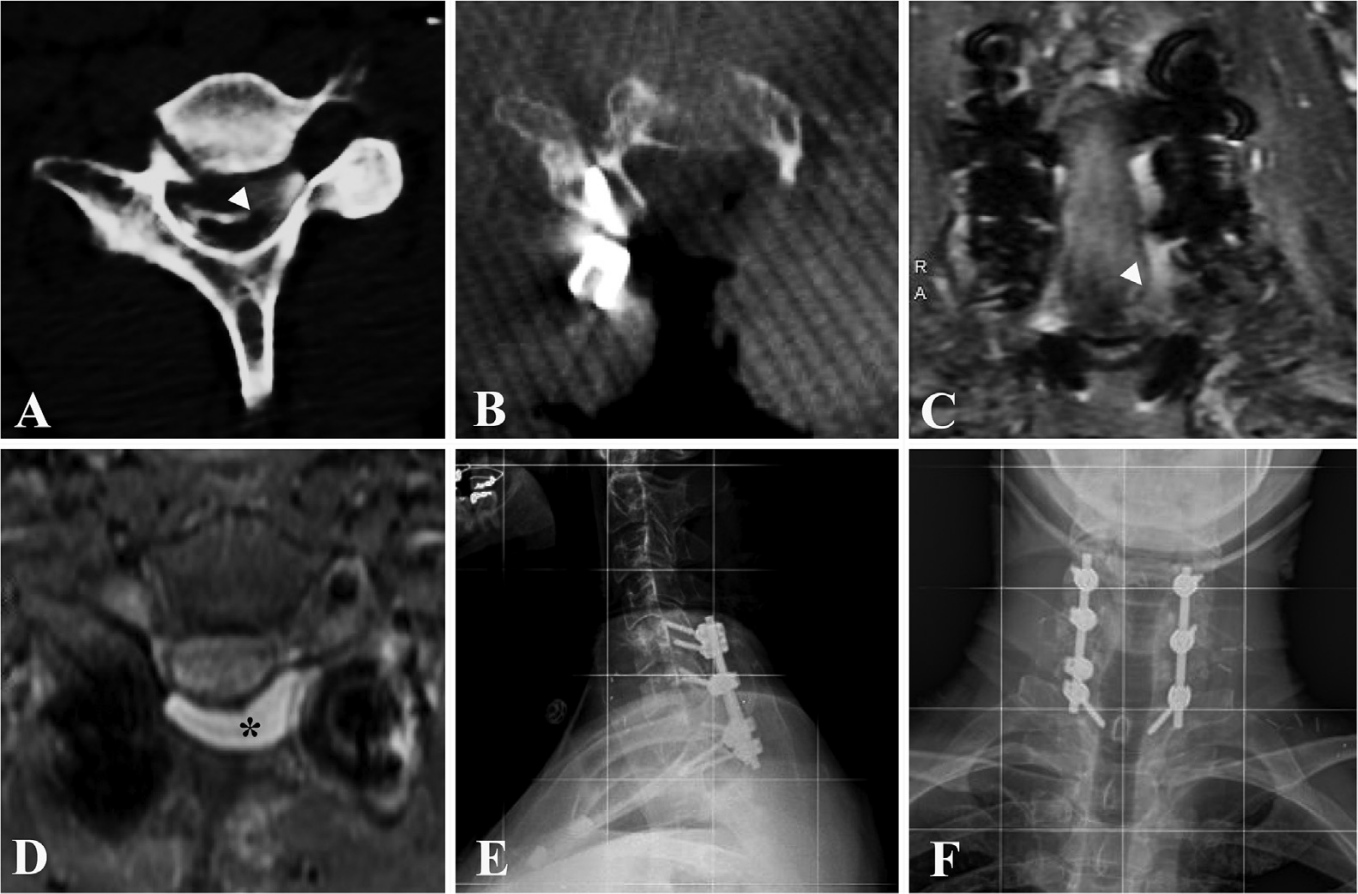
Panel A: Axial cut of a preoperative CT myelogram showing spinal cord herniation (SCH) through a dural defect after C8 nerve root avulsion (white arrowhead), and compression due pseudomeningocoele. Panel B: Axial cut of an intraoperative CT scan at the same level as panel A showing right-sided lateral mass screw, and extent of bony resection completed to allow dural repair (fascetectomy, pedicular resection). Panel C: Coronal view, T2-weighted sequence, of a postoperative MR neurogram. Instrumentation artifact and previously repaired BPAI-SCH with absence of nerve root sleeve pseudomeningocoele (white arrowhead) are visualized. Panel D: Axial cut of MR neurogram (T2 sequence) showing postoperative seroma (black asterisk), instrumentation artifact, and repaired BPAI-SCH with absence of nerve root sleeve pseudomeningocoele. Panels E and F: Postoperative lateral and anteroposterior plain XR, respectively, showing instrumentation in place.

The radiological data included in Fig. 1 are the preoperative myelogram, intraoperative CT, and postoperative follow-up imaging obtained for Case 2 presented in the accompanying article [5]. The data shown were collected and included to highlight the problem in question and demonstrate the surgical approach taken for its treatment. Furthermore, the postoperative radiological data emphasize that recognition and accurate diagnosis BPAI-SCH can lead to successful treatment of this rare phenomenon. Fig. 1, panel A is an axial cut of a preoperative CT myelogram showing spinal cord herniation through a dural defect after C8 nerve root avulsion (white arrowhead), and compression due to associated pseudomeningocoele. Panel B is an axial cut of an intraoperative CT scan at the same level as panel A showing a right-sided lateral mass screw, and the extent of bony resection completed to facilitate dural repair (fascetectomy, pedicular resection). Panel C is a coronal view, T2-weighted sequence from the postoperative MRI neurogram of Case 2. Instrumentation artifact and previously repaired BPAI-SCH with absence of nerve root sleeve pseudomeningocoele (white arrowhead) are visualized. Panel D is an axial cut from the same MR neurogram sequence showing postoperative seroma (black asterisk), instrumentation artifact, and previously repaired BPAI-SCH with absence of nerve root sleeve pseudomeningocoele. Panels E and F are lateral and anteroposterior plain XR views, respectively, of the postoperative radiograph from Case 2 with instrumentation in place. Key patient and disease demographic, clinical, radiographic, and technical features pertaining to ATSCH and BPAI-SCH identified and collected from the outlined literature review are shown in Table 1. This data was collected and included in table format to compare and contrast these two phenomena as distinct entities, and supplements such a discussion found in the accompanying article.

**Table 1:** Table contrasting anterior thoracic spinal cord herniation (ATSCH) and brachial plexus avulsion injury associated spinal cord herniation (BPAI-SCH), highlighting the two as distinct entities.

		ATSCH [[1], [2], [3], [4],^6^]	BPAI-SCH [5,[7], [8], [9], [10]]
Mean age (years; range)		Middle-older age adult (51; 21–78)	Younger-middle age adult (32.5; 18–41)
Sex		M:F 1:1.8	M (all reported cases)
Presentation		Commonly: Brown-Sequard syndrome, Paraparesis	Commonly: myelopathy, unilateral pyramidal symptoms Less commonly: Brown-Sequard and Horner's syndrome
Spinal location (dural defect)		Thoracic (ventral/lateral)	Cervicothoracic junction (lateral)
Mean symptom duration at diagnosis (years; range)		4.5 (range: 1.0–12.0)	2.0 (range: 0.25–4.0)
Mean time to presentation post-injury (years; range)			9.0 (range: 2.0–19.0)
Etiologies		Multiple: - Spontaneous - Inflammatory/erosive - Congenital - Traumatic - Iatrogenic	- Post-traumatic
Associated pathology and Operative findings		- Dural duplication - Arachnoid cyst - Spinal cord herniation - Adhesions/tethering - Syrinx - Bony erosion - Disc Herniation - Previous bony fracture	- Nerve root avulsion - Spinal cord herniation - Pseudomeningocoele - Superficial siderosis - Adhesions/tethering - Syrinx
Natural History		Variable: - Conservative treatment reported with neurological deterioration and stabilization being described	Unclear: - All reported cases treated operatively
Treatment/Dural repair techniques		Multiple - Cord release (untethering/reduction) - Dural defect widening (dural duplication present) - Direct suture (space permitting) - Dural-patch graft - Fat graft - With/without bony instrumented fusion	Multiple - Cord release (untethering/reduction) - Direct suture (if space - Dural-patch graft - With/without bony instrumented fusion
Operative outcome		- Improvement: 74% - Stabilization: 18% - Worsening: 8%	- Improvement/stabilization: all cases reported
Prognostic Factors (motor deficit improvement)		- Brown-Sequard syndrome - Cord release (untethering/reduction) - Dural defect widening (dural duplication cases)	

## 2 Experimental design, materials, and methods

An electronic medical database was utilized as part of routine follow-up care to obtain patient demographic, clinical, and radiographic information pertaining to the cases discussed in the accompanying article [5]. More specifically, postoperative radiographic imaging (MRI neurogram and plain XR) was obtained from the attending health care institution for the creation of Fig. 1 in Adobe Photoshop Creative Suite 6 software (San Jose, California, USA). Table 1 was created through literature search. PubMed, Ovid-Medline, and Google Scholar databases were searched from inception to 2019 for clinical articles in English related to spinal cord herniation. Duplicate articles were excluded; article titles and abstracts including case reports, case series, and meta-analyses were then searched and grouped into those pertaining to ATSCH and cervical SCH, which were then further refined into those dealing with BPAI-SCH. Article references were searched for inclusiveness. The articles were then examined for data pertaining to demographic, clinical, radiographic, and technical features for the two entities. With our two cases, data from the literature search were used to create a summary table comparing the two entities.

## Funding disclosure

None.

## References

[1] Darbar A., Krishnamurthy S., Holsapple J.W., Hodge C.J. (2006). Ventral thoracic spinal cord herniation: frequently misdiagnosed entity. Spine (Phila Pa 1976).

[2] Groen R.J., Middel B., Meilof J.F. (2009). Operative treatment of anterior thoracic spinal cord herniation: three new cases and an individual patient data meta-analysis of 126 case reports. Neurosurgery.

[3] Samuel N., Goldstein C.L., Santaguida C., Fehlings M.G. (2015). Spontaneous resolution of idiopathic thoracic spinal cord herniation: case report. J. Neurosurg. Spine.

[4] Summers J.C., Balasubramani Y.V., Chan P.C., Rosenfeld J.V. (2013). Idiopathic spinal cord herniation: clinical review and report of three cases. Asian J. Neurosurg..

[5] Jack A.S., Chapman J.R., Mummaneni P.V., Gerard C.S., Jacques L.G. (2020). Late Cervical Spinal Cord Herniation Resulting from Post-Traumatic Brachial Plexus Avulsion Injury.

[6] Hassler W., Al-Kahlout E., Schick U. (2008). Spontaneous herniation of the spinal cord: operative technique and follow-up in 10 cases. J. Neurosurg. Spine.

[7] DaSilva V.R., Al-Gahtany M., Midha R., Sarma D., Cooper P. (2003). Upper thoracic spinal cord herniation after traumatic nerve root avulsion. Case report and review of the literature. J. Neurosurg..

[8] Moses J.E., Bansal S.K., Goyal D. (2013). Herniation of spinal cord into nerve root avulsion pseudomeningocele: a rare cause of delayed progressive neurological deficit. Indian J. Radiol. Imag..

[9] Tanaka M., Ikuma H., Nakanishi K. (2008). Spinal cord herniation into pseudomeningocele after traumatic nerve root avulsion: case report and review of the literature. Eur. Spine J..

[10] Yokota H., Yokoyama K., Noguchi H., Uchiyama Y. (2007). Spinal cord herniation into associated pseudomeningocele after brachial plexus avulsion injury: case report. Neurosurgery.

